# Genetic Structure of the Rocky Intertidal Stalked Barnacle *Capitulum mitella* Across the Northwest Pacific and Southeast Asia: Influences of Pleistocene Climate Changes and Contemporary Oceanographic Regimes

**DOI:** 10.1093/iob/obaf042

**Published:** 2025-11-11

**Authors:** A Shahdadi, B K K Chan

**Affiliations:** Biodiversity Research Center, Academia Sinica, Taipei 115, Taiwan; Biodiversity Research Center, Academia Sinica, Taipei 115, Taiwan

## Abstract

*Capitulum mitella*, the intertidal stalked barnacle, is a common commercial seafood throughout East Asia. This study investigates the phylogeography of this species across the South China Sea and northwestern Pacific using the mitochondrial COX1 marker. Phylogenetic analyses recovered three distinct clades: (1) the NW Pacific and SE Asian clade, distributed from Vietnam, mainland China to northern Japan; (2) the South clade, found in eastern Malaysia and the Philippines; and (3) the Ryukyu clade, concentrated in Okinawa and sparsely to the Philippines. These clades likely diverged during Pleistocene glaciations, originating from different glacial refugia. Phylogeographic and demographic analyses also revealed more recent, shallower divergences within both the NW Pacific and SE Asian clade and the South clade, associated with later glacial cycles. Additionally, the genetic structure of the NW Pacific and SE Asian clade, composed of two partially isolated populations, the Northeastern and the Southwestern populations, probably resulted from thermal and salinity selections, as well as climate effects of the LGM. The NW Pacific and SE Asian clade and the South clade do not overlap in distribution within the South China Sea. In contrast, the Ryukyu clade co-occurs with the South clade in the Philippines and with the NW Pacific and SE Asian clade in Okinawa, likely facilitated by the Kuroshio Current. The broad latitudinal range of the NW Pacific and SE Asian clade (10°N to 40°N) suggests it is an eurythermal lineage capable of thriving across a wide range of temperatures.

龜足(*Capitulum mitella*), 是遍及東亞的潮間帶藤壺, 它也是常見的海產。本研究利用粒線體COX1標記, 探討龜足在南海與西北太平洋的親緣地理結構。分子系統分析顯示三個明顯分支群:1) 西北太平洋與東南亞分支群, 分布自越南、中國大陸至日本北部;2) 南方分支群, 見於東馬來西亞與菲律賓;3) 琉球分支群, 集中分布於沖繩並零星擴散至菲律賓。這些分支群可能源自更新世冰河時期不同冰川避難所的分化。親緣地理與族群動態分析進一步揭示, 在西北太平洋與東南亞支系及南方分支群內部, 存在與較晚冰期循環相關、分化程度 較淺的近緣分支。此外, 西北太平洋與東南亞分支群由東北與西南兩個部分隔離族群組成, 其遺傳結構可能源自溫度與鹽度的區隔, 以及末次盛冰期的氣候效應。西北太平洋與東南亞支系與南方支系在南海範圍內無分布重疊, 而琉球分支群在菲律賓與南方分支群共存, 在沖繩則與西北太平洋與東南亞分支群共域, 此現象可能受黑潮影響。西北太平洋與東南亞分支群橫跨10°N至40°N的廣泛緯度範圍, 顯示其為廣溫性演化支, 能適應跨溫帶至亞熱帶的寬廣溫度區間。

## Introduction

The peculiar bathymetry of the NW Pacific (including the South China Sea), with several deep-sea basins surrounded by shallow water and the complicated oceanographic regimes, positioned this region at the center of attention for biogeographic studies (e.g., Chan and Lee 2012; Ni et al. 2014). Analyses of molecular phylogeography have revealed cryptic diversity in many of the coastal species complexes, which were assumed to be a single species (lineage) across the northwestern Pacific and the South China Sea. Representatives of different taxa have been studied, and a variety of phylogeographic patterns were recovered (e.g., coastal barnacle in Wu et al. 2015; green mussel in Ye et al. 2015; mantis shrimp in Cheng and Sha 2017; and mangrove crabs in Shahdadi et al. 2022).

With sessile adult and pelagic larvae, the phylogeography of coastal barnacles is influenced by Pleistocene climate changes and sea level fluctuations as well as by present-day hydrographic characteristics such as oceanic currents and sea surface temperature (Tsang et al. 2012a, [Bibr bib65][Bibr bib65]). Differences in larval durations and tolerance against hydrographic barriers, as well as habitat preferences, have formed a variety of genetic structures among different barnacle species (Cheang et al. 2012; Shahdadi et al. 2024). Tsang et al. (2012b) discovered three distinct clades of *Chthamalus malayensis* along the West Pacific; that is, South China Sea clade distributed from the Gulf of Thailand, through Vietnam and China, to Taiwan and the Philippines; Taiwan clade, restricted the East and North coasts of Taiwan; and the Indo-Malay clade distributed from the Bay of Bengal to Eastern Malaysia (north of Borneo). Chan et al. (2007) discovered two separate clades for *Tetraclita squamosa* distributed along China, while *T. kuroshioensis* is distributed from Taiwan to Japan (Okinawa to Honshu). Cheang et al. (2012), in contrast, detected no divergence in *C. challengeri* from China to Honshu (Japan).

The monotypic stalked barnacle of the genus *Capitulum* (Gray 1825), is represented by a single species, *C. mitella* (Linnaeus 1758), commonly known as “turtle’s hand” in China, Taiwan, and Japan (kamenote in Japanese). *Capitulum mitella* is a well-known commercial seafood species and very common in East Asia, distributed widely throughout the West Pacific from the Philippines to Japan (Jones et al. 2000; Chan 2007). Because of its abundance and wide distribution, *C. mitella* is becoming a model species among stalked barnacles, and different aspects have been studied (e.g., larval morphology in Lee et al. 2000; Rao and Lin 2014; Kado et al. 2025; metamorphosis in Lin and Rao 2016; Rao and Lin 2020; and adult growth in Nakamura and Tanaka 1995). Tian et al. (2020) released its mitochondrial genome, and later its whole chromosome-level genome was also sequenced (Chen et al. 2021; Yuan et al. 2024). Three works have also tried to locally study the population genetics of *C. mitella*. Yuan et al. (2016) and Xing et al. (2023) studied this species along the Chinese coasts using the COX1 and 16S markers, respectively. Yoon et al. (2013) studied this species in South Korea using the control region. They did not detect any phylogeographic structure as their sampling regions were limited.

Previous studies in East and Southeast Asia all focused on sessile barnacles (see above), and no study has tried to investigate the phylogeography of *C. mitella* across a wider area of the West Pacific. The present study, therefore, aims to address the phylogeography of *C. mitella* in a wider geographic area from Vietnam, Eastern Malaysia, China, the Philippines, Taiwan, and the Ryukyu Islands to the northern Japanese island of Sapporo. As *C. mitella* is a sessile invertebrate, this study examines how oceanic regimes—particularly currents along the Western Pacific—shape its genetic structure through larval dispersal. Maggs et al. (2008) proposed four models describing how Quaternary glaciations have influenced the phylogeographic structure of marine species: Model I, the null model (panmixia); Model II, a latitudinal cline in allelic richness; Model III, the existence of two or more refugia-derived populations with little or no contact; and Model IV, two or more refugia-derived conspecific populations with secondary contact. This study also aims to evaluate which of these models best explains the genetic structure of *C. mitella* across the Western Pacific.

## Methods and materials

### Specimen collections and sequence preparations

**Fig. 1:**
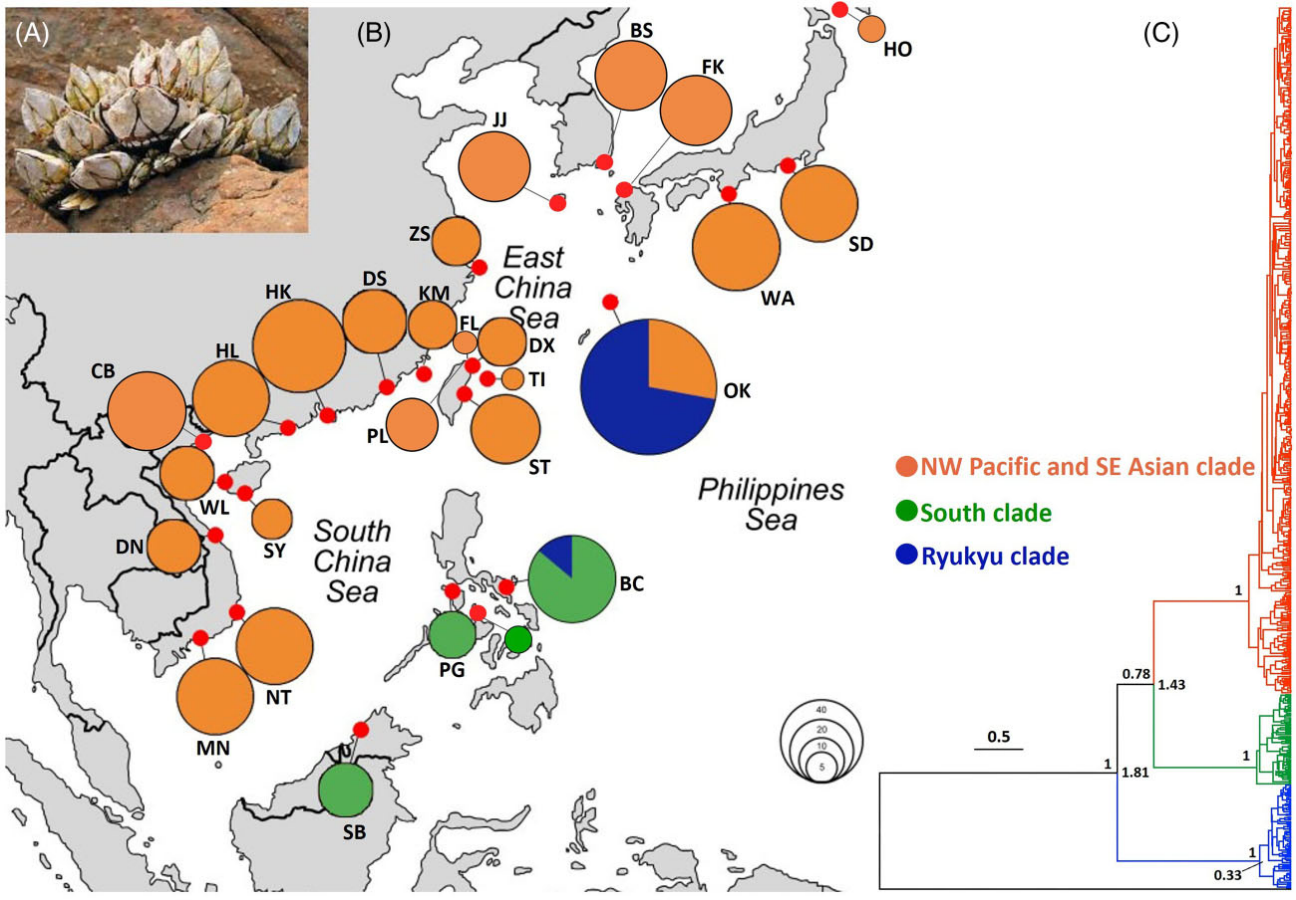
(A) Specimens of *C. mitella* in a rocky intertidal shore. (B) Map showing the sampling sites of *C. mitella* in this study, with circle size proportional to the number of sequences available from each locality. The colors represent different phylogenetic clades. For the abbreviation of the localities and geographic coordinates, see Tables 1 and S1. (C) Bayesian phylogram (constructed in BEAST 2.7.7) showing phylogenetic relationships among sequences of *C. mitella*, based on COX1 sequences (630 bp). A sequence of *Polycipes policipes* was used as an outgroup. The numbers left to the nodes are posterior probabilities, and the numbers on the right of the nodes are divergence time in million years.

Specimens of *Capitulum mitella* (Fig. 1A) were collected by hammer and chisel from crevices during low tides from 27 localities in Vietnam, China, Taiwan, Japan, Eastern Malaysia, and the Philippines (for the distribution map, material examined, and the localities see Fig. 1B, Table 1, and Table S1). The specimens were preserved in 95% ethanol prior to DNA extraction.

**Table 1:** Localities from where specimens of *C. mitella* were collected for this study with the abbreviations and number of sequences available from each locality and clade (see Fig. 1). For geographic coordinates and GenBank accession numbers see Table S1.

	Localities	Abbreviation	Total sequences	NW Pacific and SE Asian	Ryukyu	South
1	Nexus Beach, Sabah, Malaysia	SB	13	0	0	13
2	Boracay, Philippines	BY	3	0	0	3
3	Puerto Galera, Philippines	PG	16	0	0	16
4	Legazpi, Bicol, Luzon, Philippines	BC	35	0	5	30
5	Okinawa, Japan	OK	93	26	67	0
6	Hakotate, Hokkaido, Japan	HO	3	3	0	0
7	Shimoda, Japan	SD	29	29	0	0
8	Wakayama, Japan	WA	43	43	0	0
9	Shikanosima, Fukuoka, Japan	FK	17	17	0	0
10	Oryukdo wharf, Busan, South Korea	BS	24	24	0	0
11	Marodo, Jeju Island, South Korea	JJ	14	14	0	0
12	Turtle Island, Taiwan	TI	2	2	0	0
13	Shi Ti Ping, Taiwan	ST	25	25	0	0
14	Pinglang Bridge, Keelung, Taiwan	PL	14	14	0	0
15	Da Xiang Ling, Taiwan	DX	12	12	0	0
16	Kinmen Island, Taiwan	KM	12	12	0	0
17	Fulong, Taiwan	FL	3	3	0	0
18	Zhoushan, China	ZS	12	12	0	0
19	Dongshan, China	DS	23	23	0	0
20	Hong Kong, China	HK	49	49	0	0
21	Hoi Ling, China	HL	32	32	0	0
22	Sanya, Hainan, China	SY	14	14	0	0
23	Wanling, Hainan, China	WL	15	15	0	0
24	Cat Ba, Vietnam	CB	30	30	0	0
25	Danang, Vietnam	DN	15	15	0	0
26	Nha Trang, Vietnam	NT	29	29	0	0
27	Muine, Vietnam	MN	29	29	0	0

Genomic DNA was isolated from part of the soft body using the QIAGEN (Germany, Hilden) DNeasy^®^ Blood & Tissue Kit, following the manufacturer’s protocol. A partial segment of a mitochondrial gene, the protein-coding gene cytochrome c oxidase subunit 1 (COX1) (the Folmer region, also known as the barcode region, Hebert et al. 2003) was amplified by polymerase chain reaction (PCR). The PCRs were performed using the universal primers, LCO1490 (5ʹ-GGTCAACAAATCATAAAGATATTG G-3ʹ) (forward) and HCO2891 (5ʹ-TAAACTTCAGGG TGACCAAAAAATCA-3ʹ) (reverse) (Folmer et al. 1994). Each PCR reaction was performed using 12.5 μL Master Mix (PowerAmp 2× PCRmix-Green, BIOMAN SCIENTIFIC, Taipei, Taiwan), 0.5 μL of each primer (10 μM), 1 μL of template DNA, and 10.5 μL of distilled H2O. The PCRs were conducted in a DNA Engine Thermal Cycler (C1000 Touch, Bio-Rad, Richmond, CA, USA) with the following profiles: initial denaturation step for 5 mins at 95°C; 35 cycles of 30 s at 95°C for denaturing, 60 s at 48.7°C for annealing, 45 s at 72°C for extension; and a final extension step of 7 mins at 72°C. The PCR products were checked by electrophoresis on 1% agarose gel in 1× TAE buffer. PCR products were outsourced to Genomics BioSci & Tech Ltd (Taiwan, Taipei) for Sanger sequencing. PCR products were sequenced using the forward primers. New sequences were submitted to GenBank (https://www.ncbi.nlm.nih.gov/) and are available under accession numbers given in Table S1. Sequences were proofread using Chromas Lite (v. 2.1.1) (Technelysium Pty Ltd, Queensland, Australia); the primer regions and adjacent bases with low quality scores (Phred scores lower than 20) were removed. The sequences were aligned with ClustalW (Thompson et al. 1994) implemented in BioEdit 7.0.5 (Hall 1999).

### Phylogenetic analyses

To infer phylogenetic relationships among sequences of *C. mitella*, a Bayesian inference was conducted in BEAST 2.7.7 (Bouckaert et al. 2019). The FASTA file of the COX1 alignment was used as input, and a Yule process and a strict clock were used as priors for the tree and clock models, respectively. Markov Chains were run for 10 million generations, sampling every 1000 iterations and discarding the first 10% as burn-in. The remaining 9000 trees were used to calculate the maximum clade credibility tree in Tree Annotator (part of the BEAST package). The best evolutionary models were obtained using ModelFinder (Kalyaanamoorthy et al. 2017) through the IQ-TREE web server based on Bayesian information criterion (BIC) scores, and were used in the phylogenetic analysis. The best-fit model according to BIC scores was TN + F + G4. Wares (2001) compared sister species of *Microeuraphia* across the Panamanian Isthmus and recovered a divergence rate of 3.1% (substitution rate = 1.55%) per million years (my) for COX1 for barnacles. We used this rate to estimate the divergence time among sequences of *C. mitella.* A sequence of *Pollicipes pollicipes* Gmelin, 1791 (accession number = KF484186) was used as an outgroup.

The software Assemble Species by Automatic Partitioning (ASAP) (https://bioinfo.mnhn.fr/abi/public/asap/asapweb.html) (Puillandre et al. 2012) was used for species delimitation with their default options, using their online servers. The aligned FASTA file of the COX1 (without the outgroup) was used as the input, and the simple p-distance was selected as the method for the calculation.

### Phylogeography and demography

Phylogenetic analyses recovered three well-defined clades: the NW Pacific and SE Asian clade, the Ryukyu clade, and the South clades (see Results). We used MEGA X (Kumar et al. 2018) to calculate mean genetic distances (p-distance) between the three clades.

To obtain a better resolution of the genetic relationships among sequences and localities within each clade, a TCS haplotype network (Templeton et al. 1992) was built for each clade separately, using the software PopART (Leigh and Bryant 2015).

To further detect and visualize genetic affinities among the localities in the NW Pacific and SE Asian clade and the South clade (with sequences from more than two localities for each clade), principal component analyses (PCoA) were performed in GenAlEx 6.5 (Peakall and Smouse 2012). The fixation index, Φst, between localities was calculated with ARLEQUIN 3.1 (Excoffier and Lischer 2010) using 1000 permutations. Localities with fewer than 10 sequences (see Table 1) were not included in these statistical analyses (PCoA and Φst) due to low sample size. However, to run PCoA, we needed at least four localities. Thus, for the South clade, we included Boracay (BY) with only 3 sequences.

Based on haplotype network, PCoA and Φst values, the localities of the NW Pacific and SE Asian clade were divided into two populations (Southwestern and Northeastern, see the results). These analyses also detected two genetic groups (hereafter called Western and Eastern) in the South clade. These divisions were considered for the following population analyses. To identify genetic differentiations among localities within the NW Pacific and SE Asian clade and South clade, analyses of molecular variance (AMOVAs) were performed in GenAlEx 6.5 using 999 permutations. These AMOVA analyses were performed once without categorizing localities into populations or groups, and once assuming two populations for the NW Pacific and SE Asian clade and two regions for the South clade.

The clade of Ryukyu was not included in PCoA, Φst, and AMOVA, as most sequences of this clade were only from Okinawa, and the second locality, Bicol (BC), includes only 5 sequences of this clade.

To detect possible isolation by distance in the NW Pacific and SE Asian clade (more widespread clade), a Mantel test (Mantel 1967) was performed in GenAlEx 6.5 with 999 permutations, using geographic coordinates and direct distances. The Mantel test was conducted once for all localities in this clade, but to exclude isolative effects of oceanographic factors between the two populations in the NW Pacific and SE Asian clade, a second Mantel test was conducted for localities only within the Southwestern population of this clade.

To trace population size changes in the three clades, we analyzed the distribution of pairwise differences (mismatch distribution) (Rogers and Harpending 1992) in ARLEQUIN 3.5 with a model of sudden demographic expansion and spatial expansion for expected values, and graphs were created in Microsoft Excel 2013. The mismatch distribution analyses were conducted for populations of the NW Pacific and SE Asian clades and the genetic groups of the South clade separately. We also used a Bayesian Skyline Plot (BSP) (Drummond et al. 2006) in BEAST 2.7.7 to assess how mtDNA effective population size changed through time for the two populations of the NW Pacific and SE Asian clade. We used HKY + F and TN + F + I (for Southwestern and Northeastern populations, respectively) as the models of nucleotide substitution, suggested as Best-fit models according to BIC scores by ModelFinder (Kalyaanamoorthy et al. 2017). We used a substitution rate of 1.55% per million years as suggested by Wares (2001).

Genetic diversity indices were also calculated for each clade and the populations (genetic groups) within each clade in DnaSP v6 (Rozas et al. 2017).

## Results

In total, 606 COX1 sequences were obtained for specimens of *C. mitella* from 27 localities in the South China Sea to the Northwest Pacific, with the final alignment of 630 base pairs long (see Tables 1 and S1) (accession numbers PX472105-PX472711).

### Phylogeny

Phylogenetic analysis recovered three well-supported distinct clades for the sequences of *C. mitella* around South China Sea and Northwest Pacific. The most widespread clade, which is named as the NW Pacific and SE Asian, is distributed from south to north of Vietnam, southern China coasts from Hainan Island and Hong Kong to Zhoushan, on the southern side of the Yangtze River estuary, east to north coasts of Taiwan, South Korea, Okinawa, Honshu to Sapporo islands in Japan. The second clade, which is named here as the South clade, is distributed in the north of Borneo (Sabah, Eastern Malaysia) to the Philippines islands of Mindoro, Boracay, and the southeastern Luzon (Bicol). The third clade, the Ryukyu clade, is mainly distributed in Okinawa, with five sequences in the southeastern Luzon (Bicol) (Figs. 1B, C, and S1). The three clades are highly supported with a posterior probability of 1. The NW Pacific and SE Asian clade and the South clade are sister groups in the COX1 tree, but not well supported. Mean genetic p-distances were 0.08 between NW Pacific and SE Asian clade and the South clade, and were 0.09 between both of these clades and the Ryukyu clade. Species delimitation analyses recovered three molecular operational taxonomic units (mOTUs) corresponding to the three phylogenetic clades with the best score (asap-score = 1.50; *P*-val = 3.37e-0.4; *W* = 3.37e-04; Threshold dist. = 0.035885). The molecular clock analysis estimated the first divergence among the three *C. mitella* clades to have occurred around 1.8 million years ago (mya) (median; 95% HPD: 1.18–2.59 mya), and the second divergence around 1.43 mya (95% HPD: 0.92–2.05 mya).

### Phylogeography

#### NW Pacific and SE Asian clade

**Fig. 2:**
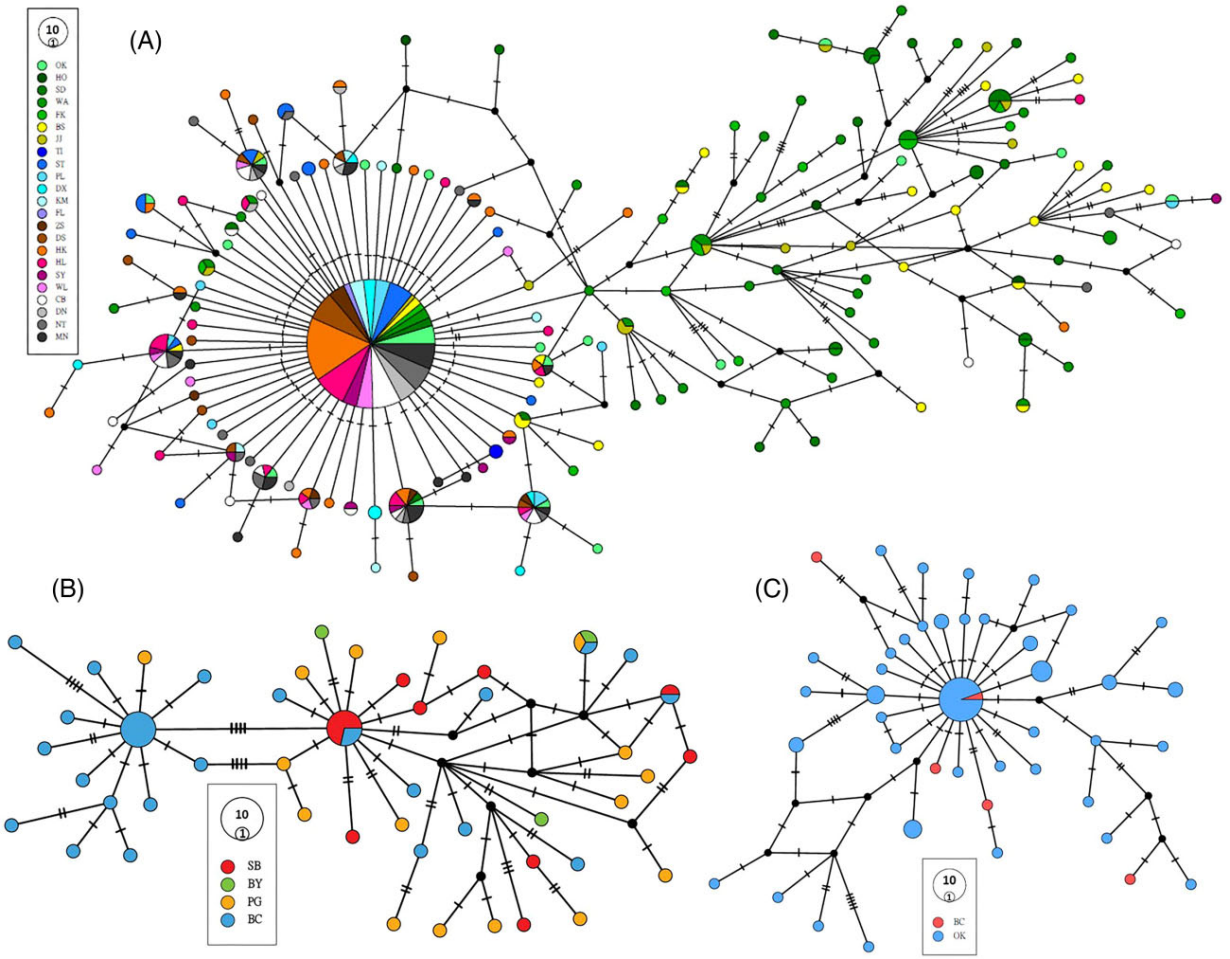
TCS haplotype networks of 630 bp of COX1 gene for sequences of *C. mitella*, constructed in PopArt 1.7. (A) For the NW Pacific and SE Asian clade (427 sequences from 23 localities). (B) For the South clade (62 sequences from 4 localities). (C) For the Ryukyu clade (72 sequences from 2 localities).

In the NW Pacific and SE Asian clade, of 472 sequences from 23 localities, 166 haplotypes, including 128 singletons, were recovered (Table 2, Fig. 2A). The most common haplotype includes 193 sequences (40%) from 21 localities in southern Vietnam to South Korea and the Japanese Island of Honshu. Some other haplotypes also include sequences from several different localities. The haplotype network of the NW Pacific and SE Asian clade (Fig. 2A) consists of two distinct populations. One population has a star-like shape with the common haplotype in the middle, surrounded by many other haplotypes with a single mutation. Most sequences from South Korea and Japan (except Okinawa), in contrast, are more distributed and distant from the main haplotype and form the second population.

**Table 2:** Pairwise Φst values among the localities of the NW Pacific and SE Asian clade of *C. mitella* calculated in ARLEQUIN 3.5.2.2.

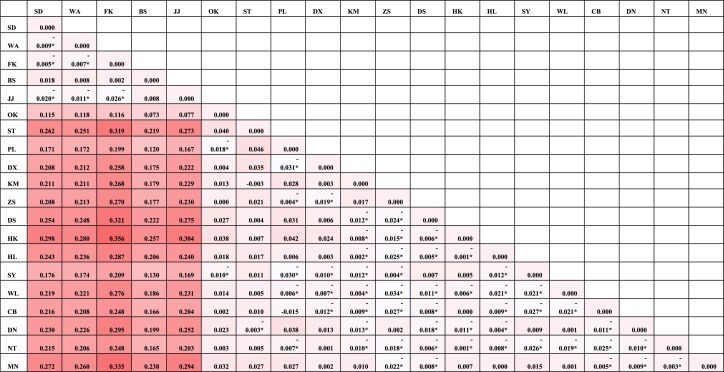

(*: *P* > 0.05, otherwise *P* < 0.05) (colors are red for high and white for low values).

**Fig. 3:**
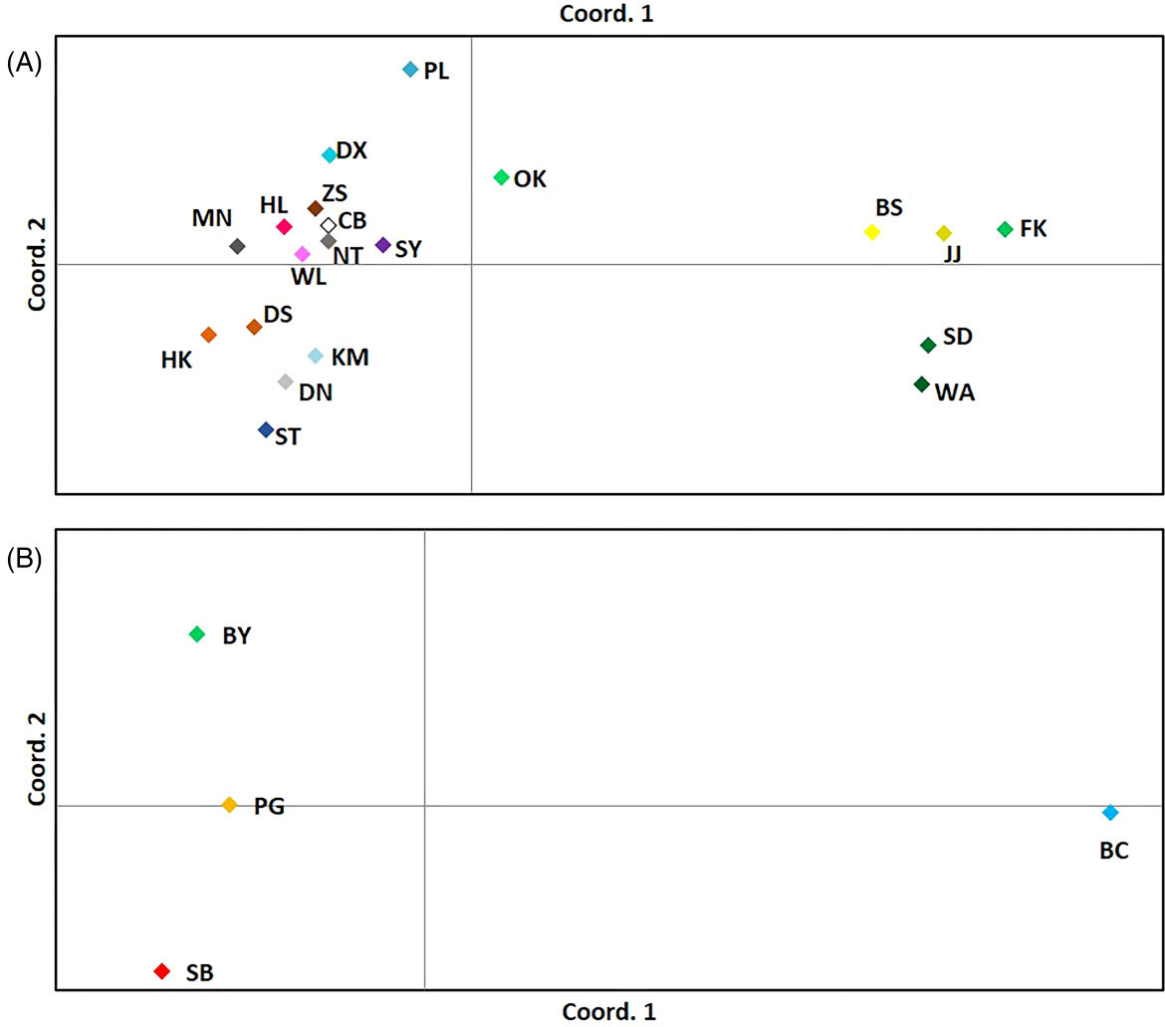
PCoA plots of COX1 sequences of *C. mitella*, constructed in GenAlex 6.5. (A) For 20 localities in the NW Pacific and SE Asian clade. (B) For 4 localities in the South clade. In GenAlEx, we used the PhiPT matrix generated from AMOVA (PhiPTP sheet) to calculate Principal Coordinates (Coordinates 1, 2, and 3). Coordinates 1 and 2 were used to plot the genetic relationships among localities. The color codes correspond with colors in the haplotype network.

Plots of PCoA (Fig. 3A) also recovered two populations for the NW Pacific and SE Asian clade. The Southwestern population includes sequences of 18 localities from south of Vietnam, China, Taiwan, to Okinawa. The Northeastern population includes 5 localities from South Korea to Honshu (Japan) (i.e., BS, JJ, FK, SD, and WA, see Fig. 1B for localities in the map; HO was not included as only three sequences were available).

The values of Φst between localities in the NW Pacific and SE Asian clade (Table 3) are generally low (lower than 0.01). These values are higher when the localities of the Northeastern population (i.e., BS, JJ, FK, SD, and WA) are compared with localities in the Southwestern population (the cells with red color in Table 2). The Φst values are slightly lower between localities of the Northeastern population and Okinawa, compared to other localities in the Southwestern population.

**Table 3:** Pairwise Φst values among the localities of the South clade of *C. mitella* calculated in ARLEQUIN 3.5.2.2.

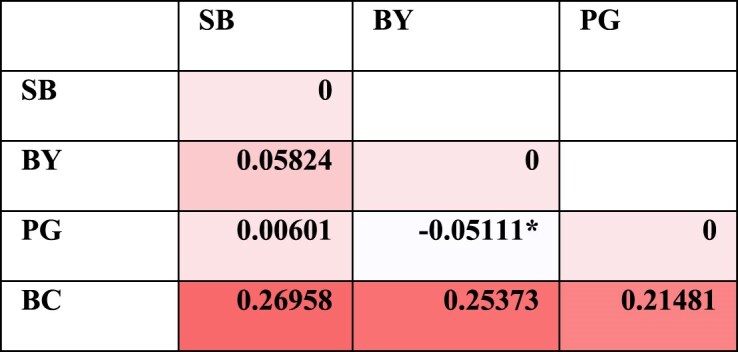

(*: *P* > 0.05, otherwise *P* < 0.05) (colors are red for high and white for low values).

**Fig. 4:**
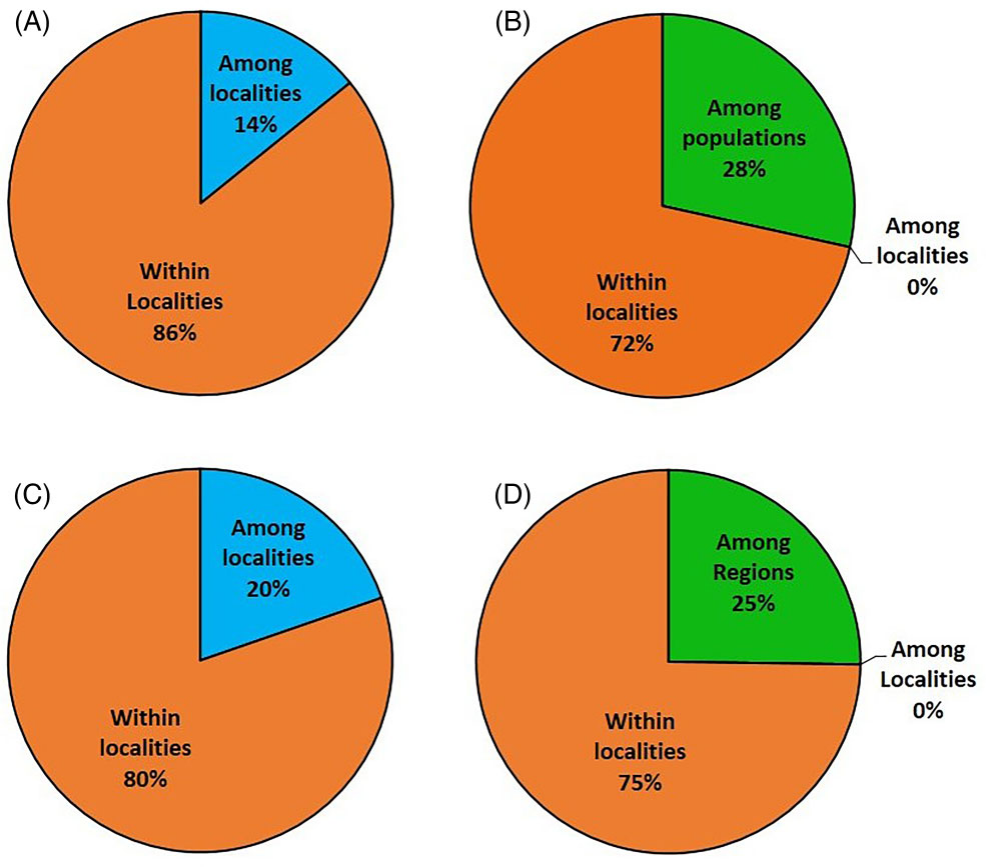
Pie graphs showing AMOVA results for COX1 sequences of *C. mitella*, calculated in ARLEQUIN 3.5. (A) For the NW Pacific and SE Asian clade, assuming 20 localities, and no population was specified. (B) For the NW Pacific and SE Asian clade, assuming 20 localities, distributed in 2 populations (Southwestern and Northeastern). (C) For the South clade, assuming 4 localities and no region was specified. (D) For the South clade, assuming 4 localities, distributed in 2 regions (Eastern and Western).

In the AMOVA, 20 localities of the NW Pacific and SE Asian clade were included (see method). In the initial analyses, we did not sort localities into populations, and 86% of the variance was from within localities and 14% was from among localities (Fig. 4A). When we sorted the localities into the two populations (see above), 72% of the variance was from within localities, 28% from among populations, and no variance was detected from among localities in each population (Fig. 4B).

**Fig. 5:**
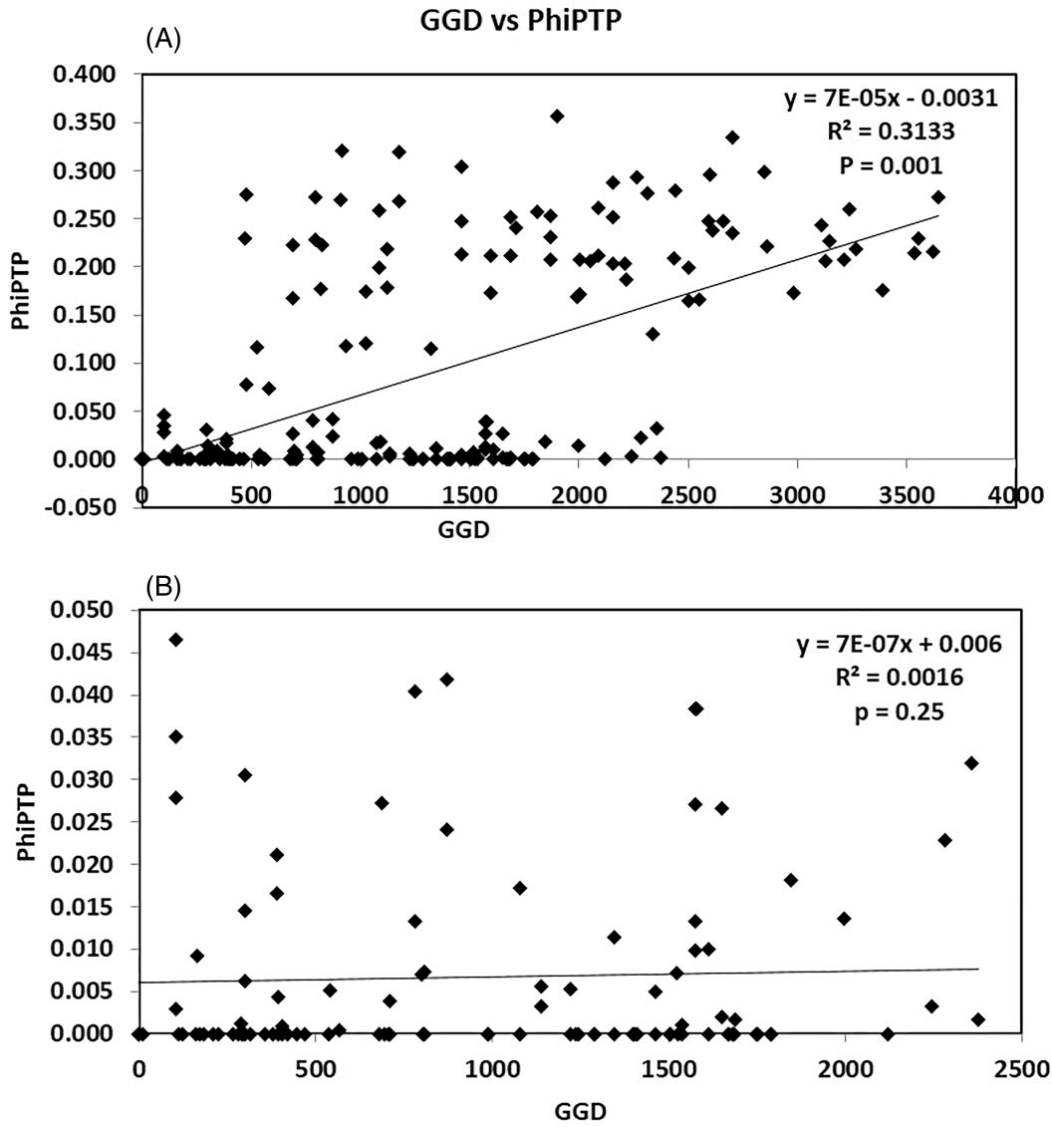
Relationship between geographical and genetic distances in *C. mitella* of the NW Pacific and SE Asian clade based on COX1 sequences. The scatterplot shows the correlation between pairwise geographical distances and pairwise genetic distances (PhiPTP). (A) For 20 localities of the NW Pacific and SE Asian clade. (B) For 15 localities of the Southwestern population of the NW Pacific and SE Asian clade. The solid line represents the linear regression fit. The Mantel test, conducted in GenAlEx.

Mantel test recovered a weak relationship between geographic and genetic distances (*R*^2^ = 0.031; *P* = 0.001) when including all localities of the NW Pacific and SE Asian clade (Fig. 5A). The relationship was much weaker when we conducted the Mantel Test only for localities of the Southwestern population (the localities of Northeastern populations, JJ, BS, FK, WA, and SD were excluded) (*R*^2^ = 0.0016; *P* = 0.252) (Fig. 5B).

#### South clade

The haplotype network of the South clade (Fig. 2B) recovered 47 haplotypes (43 singletons) distributed into two distinct genetic groups. One group is mainly distributed in BC (Bicol in the southeast of Luzon), with a single sequence from PG (Mindoro, Puerto Galera). The second group is more widely distributed in all four localities from BC (Bicol) to SB (Sabah, Eastern Malaysia).

Plots of PCoA (Fig. 3B) also confirmed the isolation of BC (Bicol) and more affinity among the other three localities (BY, PG, and SB). Congruently, the Φst values between BC (Bicol) and other localities are higher than this value among SB, BY, and PG (Table 3).

For the South clades, AMOVA was also conducted twice. When no region was specified, 80% of variance was from within localities and 20% of variance was from among the 4 localities (Fig. 4C). When regions were specified, BC as one region, and other three localities (PG, BY, and SB) as another region, 75% of variance was from within localities, 25% among regions, and no variance from among localities within each region (Fig. 4D).

#### Ryukyu clade

The haplotype network of the Ryukyu clade (Fig. 2C) recovered 43 haplotypes out of 72 sequences from two localities (Okinawa and Bicol), including 34 singletons. The most common haplotype includes 18 sequences (25%) from both localities. Most singletons were positioned around the most common haplotypes with one or two mutations.

### Demography

**Fig. 6:**
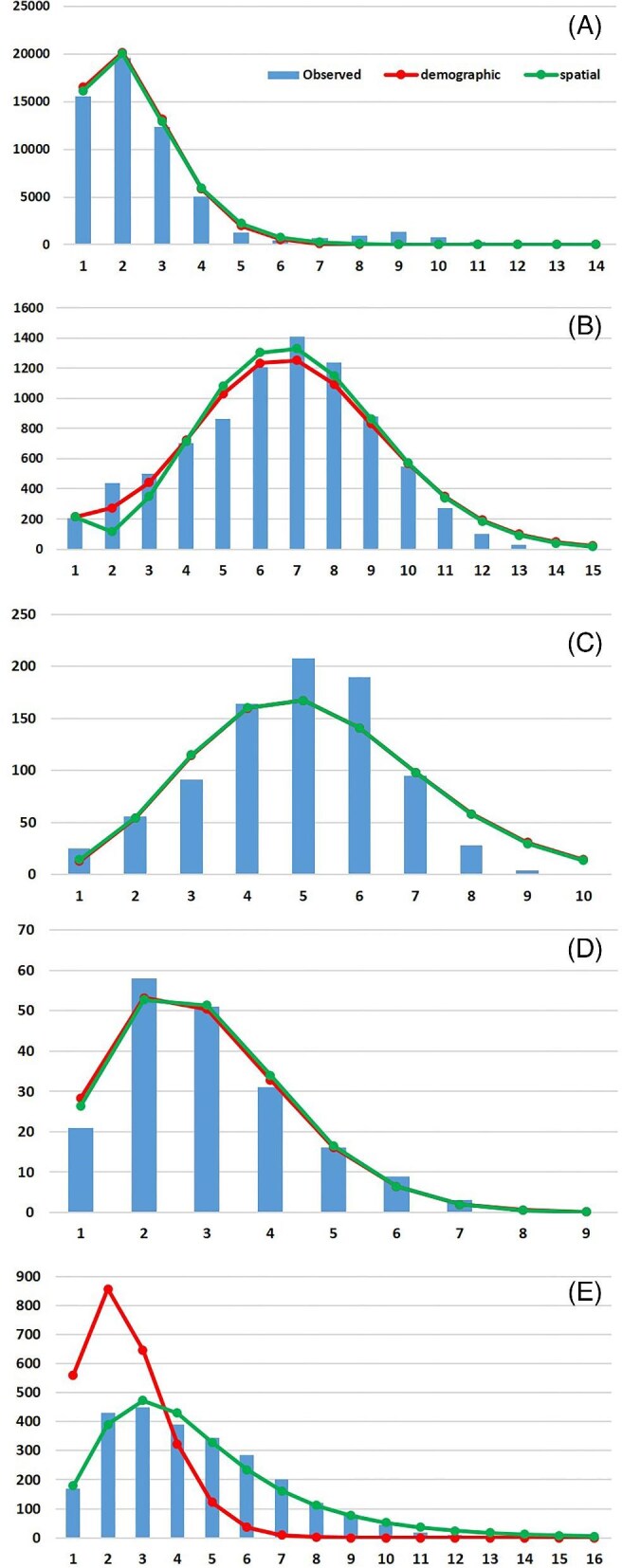
Mismatch distribution (observed values = bars, and expected values under a sudden demographic expansion model and a spatial expansion model) of COX1 sequences of *C. mitella*. The distributions were calculated in ARLEQUIN 3.5 and plotted in Excel 2013. (A) For the Southwestern population of the NW Pacific and SE Asian clade. (B) For the Northeastern population of the NW Pacific and SE Asian clade. (C) For the Western group of the South clade. (D) For the Eastern group of the South clade. (E) For the Ryukyu clade. The *x*-axis represents pairwise differences, the *y*-axis indicates frequency.

**Fig. 7:**
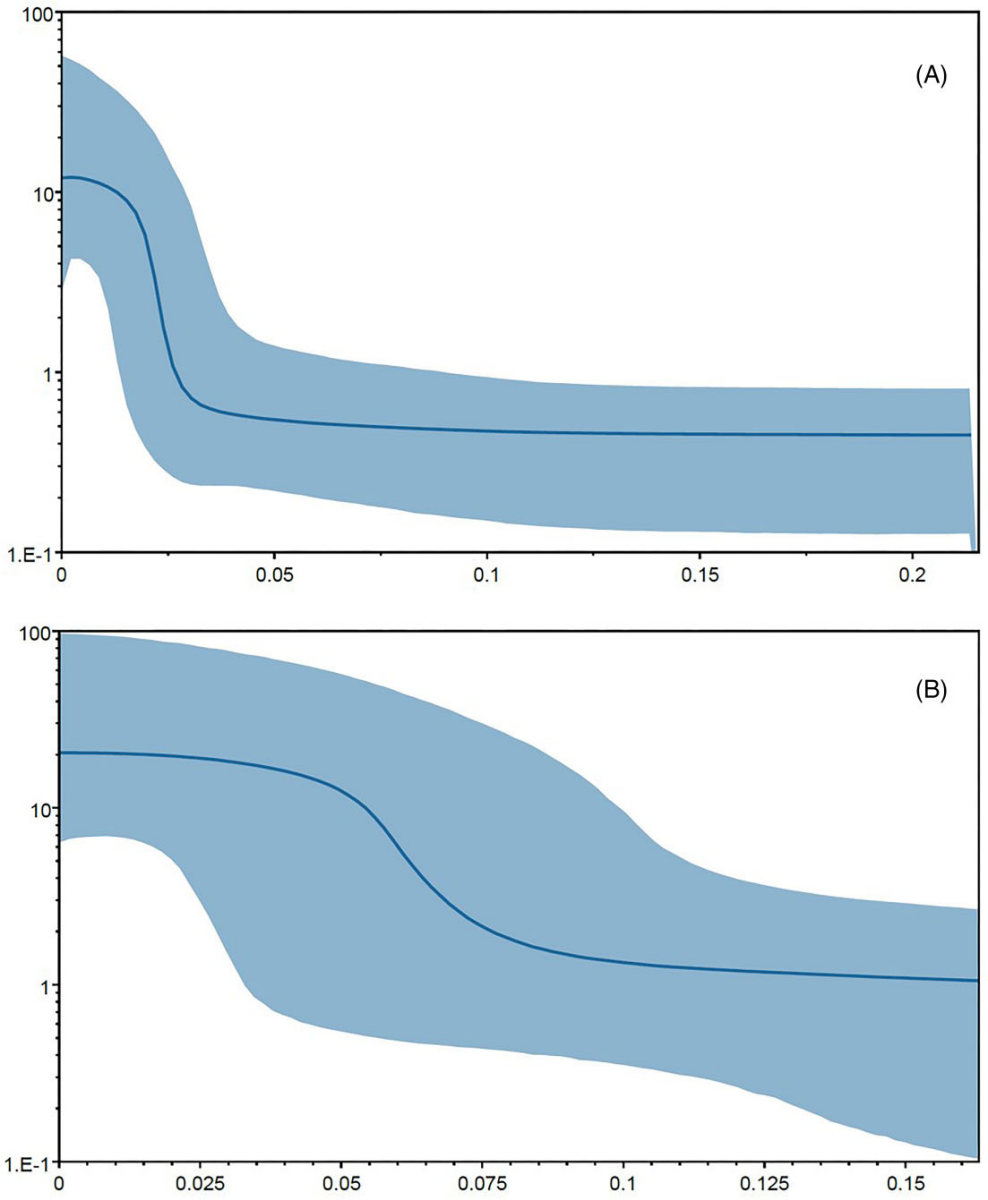
BSPs showing changes in effective population size of *C. mitella* based on COX1 sequences. (A) For the southwestern population of the NW Pacific and SE Asian clade. (B) For the Northeastern population of the NW Pacific and SE Asian clade. Molecular clock rate set at 1.55% per million years. The *x*-axis represents time before present (in million years ago); the *y*-axis indicates the effective population size (Ne) and generation length on a log scale. The central curve represents the median of the parameter Ne, proportional to the effective population size, while the shaded area shows the 95% confidence interval.

Mismatch distribution of both populations in the NW Pacific and SE Asian clade (Southwestern and Northeastern) showed a unimodal pattern and congruency between observed values and expected values under both models of sudden demographic expansion and spatial expansion (Fig. 6A, B). The low values of Harpending’s Raggedness index (*Hr* < 0.05) and high *P* values (*P* > 0.5) (Table 4) confirm recent expansion in both populations. The Tau value is lower in the Southwestern population compared to the Northeastern population (Table 4), which indicates a more recent expansion in the Southwestern population. BSPs (Fig. 7) also recovered a sudden expansion in both populations of the NW Pacific and SE Asian clade. Based on BSP graphs, the expansion in the Southwestern population happened during and after the last glacial maximum (LGM) around 0.03–0.015 my ago, while in the Northeastern population, the expansion happened earlier, around 0.08 to 0.05 million years ago.

**Table 4:** The Tau values, sum of squared deviation (SSD), and Harpending’s raggedness index (Hr) under demographic (dem) and spatial expansion model from mismatch distribution (constructed in ARLEQUIN 3.1) for the 3 clades and populations (groups) of *C. mitella*.

		Tau		SSD		Hr	
Clade	Populations/group	Dem	Spatial	Dem	Spatial	Dem	Spatial
NW Pacific and SE Asian	Southwestern	1.342	0.935	0.00201921 (*P* = 0.384)	0.00185621 (*P* = 0.282)	0.04044324 (*P* = 0.705)	0.04044324 (*P* = 0.822)
	Northeastern	6.273	5.884	0.00196846 (*P* = 0.471)	0.00314879 (*P* = 0.302)	0.00941112 (*P* = 0.697)	0.00941112 (*P* = 0.839)
	**Total**	7.973	0.309	0.00855404 (*P* = 0.827)	0.00643661 (*P* = 0.679)	0.01598332 (*P* = 0.950)	0.01598332 (*P* = 0.908)
South	Western	4.188	4.187	0.00874673 (*P* = 0.050)	0.00874419 (*P* = 0.0440)	0.03221412 (*P* = 0.132)	0.03221412 (*P* = 0.139)
	Eastern	1.930	1.941	0.00220931 (*P* = 0.821)	0.00217226 (*P* = 0.743)	0.05908587 (0.498)	0.05908587 (0.503)
	Total	5.008	4.166	0.00110889 (*P* = 0.805)	0.00193129 (*P* = 0.612)	0.00907301 (*P* = 0.860)	0.00907301 (*P* = 0.882)
Ryukyu	Total	1.498	1.498	0.08527664 (*P* = 0.000)	0.00126150 (*P* = 0.731)	0.01450513 (*P* = 0.998)	0.01450513 (*P* = 0.797)

Similar patterns of mismatch distributions, characterized by a unimodal curve, low *Hr* value, and high *P* value, were observed for the two genetic groups in the South clade (Eastern and Western) (Fig. 6C, D) and the Ryukyu clade (Fig. 6E) (Table 4).

### Genetic diversity

Among the three clades, the South clade shows higher genetic diversity (e.g., *hd* = 0.976) than the Ryukyu clade (*hd* = 0.933) and the NW Pacific and SE Asian clade (*hd* = 0.8294). Within the NW Pacific and SE Asian clade, the Northeastern population (South Korea to Japan) shows higher genetic diversity (*hd* = 0.976) compared to the Southwestern population (Okinawa to Vietnam) (*hd* = 0.733). In the South clade, the Western group shows higher genetic diversity (*hd* = 0.971) than the Eastern group (*hd* = 0.889) (Table 5).

**Table 5:** Diversity indices (calculated in DnaSP v6) for the 3 clades and populations (groups) of *C. mitella*.

Clade	Populations/group	*n*	*h*	*S*	*hd*	π
NW Pacific and SE Asian	Southwestern	342	88	90	0.733	0.00269
	Northeastern	130	86	80	0.976	0.00879
	total	472	166	117	0.8294	0.00509
South	Western	42	33	43	0.971	0.00610
	Eastern	20	14	18	0.889	0.00323
	Total	62	47	59	0.976	0.00821
Ryukyu	Total	72	43	55	0.933	0.00547

*n*, number of sequences; *nh*, number of haplotypes; *S*, number of polymorphic (segregating) sites; *hd*, haplotype (gene) diversity; and π, nucleotide diversity.

## Discussion

Our phylogenetic analysis revealed that the common and well-known stalked barnacle *C. mitella*, widely distributed across the Indo-West Pacific, is in fact a species complex comprising at least three distinct lineages distributed along the northwestern Pacific and the South China Sea. Species delimitation analyses identified three mOTUs, with genetic p-distances of 0.8–0.9%, which exceed those observed between many recognized species of invertebrates (Hebert et al. 2003), including crustaceans (Matzen da Silva et al. 2011; Chu et al. 2015) and barnacles (Wares 2001; Pitombo and Burton 2007). A similar tripartite phylogeographic structure in the northwestern Pacific has also been reported for other rocky intertidal barnacles, such as *Chthamalus moro* and *Ch. malayensis* (Tsang et al. 2012b; Wu et al. 2015).

The Pleistocene (2.4–0.011 mya), particularly from the Late Calabrian onward, was marked by pronounced climatic fluctuations and multiple major glacial cycles that profoundly reshaped global ecosystems (Richmond and Fullerton 1986; Watanabe et al. 2023). The phylogeographic structures of many marine species, especially those distributed across East and Southeast Asia, were strongly influenced by these climatic oscillations (Hewitt 2000; Woodruff 2010). Based on our estimated divergence times (2.52–0.92 mya), the separation among the three clades of *C. mitella* likely occurred during the major glacial periods of Calabrian. During glacial maxima, sea levels dropped substantially, leading to the isolation of refugial populations in deeper basins surrounded by emergent shallow seas. In the NW Pacific, likely refugia included the Sea of Japan, South China Sea, Sulu Sea, Okinawa archipelago, and Philippine Sea (Wang 1999; Voris 2000). Based on the present-day distributions of the three clades, the Ryukyu clade likely originated from the Okinawa archipelago. Okinawa appears to be a glacial refugia with many endemic marine species. The insular barnacle *Ch. moro* also has a Ryukyu lineage, distinct from the other SE Asian lineages (Wu et al. 2015). Currently, the Ryukyu clade co-occurs with the South clade in southeastern Luzon and with the NW Pacific and SE Asian clade in Okinawa. The present distributions of these three clades are likely the result of spatial expansions during interglacial periods, consistent with Model IV of Maggs et al. (2008), which describes secondary contact among refugia-derived populations.

The South clade of *C. mitella*, along with the NW Pacific and SE Asian clade, exhibit a broader geographic distribution, making it difficult to pinpoint a precise origin. The two clades currently show no overlap in their distributions and likely originated from different glacial refugia—the South clade probably from a southern refugium including the marginal seas in the Philippine archipelago (Voris 2000), and the NW Pacific and SE Asian clade from a northern one, a pattern that corresponds with Model III of Maggs et al. (2008). Although both clades occur in the South China Sea, the NW Pacific and SE Asian clade and the South clade exhibit non-overlapping distributions, suggesting that contemporary oceanographic barriers continue to limit gene flow between them. A similar pattern of phylogeographic separation between the southern and northern regions of the South China Sea has been reported in a coastal crab species complex (Shahdadi et al. 2022). The South China Sea Throughflow, which moves from the Luzon Strait through the Karimata Strait to the Java Sea (Liu 2013; Qin et al. 2016; Wei et al. 2019), may contribute to reduced genetic connectivity between northern and southern populations by disrupting reciprocal larval dispersal. However, the divergence between these two clades dates back to the Middle Pleistocene (see above), when the Karimata Strait was closed during major glacial periods and the coastline was continuous between Borneo and Vietnam (Voris 2000). Therefore, additional historical or ecological factors must have contributed to the interruption of genetic connectivity between northern and southern populations in the South China Sea.

The genus *Capitulum* has a long evolutionary history dating back to the Late Cretaceous and was once widely distributed from the Pacific to Europe (Veselská et al. 2015). The Pleistocene glacial periods, which led to population subdivision in *C. mitella* across the western Pacific, may also have contributed to the extinction of other *Capitulum* species in other oceans. The western Pacific, where three distinct clades of *C. mitella* were identified, is renowned for its exceptional marine biodiversity—driven by multiple mechanisms, including the “Center of Origin” and “Center of Accumulation” hypotheses (e.g., Briggs 2000; Carpenter et al. 2011; Shahdadi et al. 2025a). This region has also been proposed as a “Center of Survival,” as many extant species originated from Miocene cladogenetic events, facilitated by its relative environmental stability and the persistence of suitable coastal habitats during the Pleistocene glacial cycles (Barber and Bellwood 2005; Pellissier et al. 2014).

The Ryukyu clade of *C. mitella* is distributed from the eastern coast of the Philippines (Luzon) to Okinawa, while the NW Pacific and SE Asian clade is found across Taiwan, Okinawa, and the eastern coast of Honshu. Genetic connectivity along the eastern margin of *C. mitella* in the northwestern Pacific—spanning Luzon, Taiwan, Okinawa, and Honshu—is likely facilitated by the Kuroshio Current (Liu 2013; Rudnick et al. 2015), which promotes larval dispersal along the Batanes and Ryukyu Island chains, acting as stepping stones (Tsang et al. 2013; Shahdadi et al. 2025b). However, it remains unclear whether the geographically overlapping lineages form hybrids or are reproductively isolated due to biological or ecological mechanisms. Tsang et al. (2008) reported that different lineages of *Ch. malayensis* and *Tetraclita* spp. occupy distinct vertical zones in the intertidal habitat, resulting in ecological isolation even when they occur sympatrically (see also Appelbaum et al. 2002; Chan et al. 2008).

The NW Pacific and SE Asian clade of *C. mitella* is the most widespread, ranging from southern Vietnam to Sapporo in northern Japan. This extensive distribution spans two marine provinces—the Central Indo-Pacific and the Temperate Northern Pacific—and several ecoregions defined by Spalding et al. (2007). It also encompasses three zoogeographic zones according to Briggs (1995): the Tropical Zone, the Japan Warm Temperate Zone, and the Oriental Zone. A comparable distribution is seen in the chthamalid barnacle *Ch. challengeri*, which ranges from Kinmen Island (Taiwan outlying island) in the South China Sea to Sapporo in Japan, covering both the Japan Warm Temperate and Oriental zones (Cheang et al. 2012). The broad latitudinal range of *C. mitella*—from approximately 10°N to 40°N—indicates that it is a eurythermal species capable of tolerating a wide thermal gradient. This thermal flexibility is particularly notable given its intertidal habitat, where it is exposed to both sea surface temperatures and extreme air temperatures, from tropical summer highs around 35°C to temperate winter lows reaching –10°C (Hokkaido). *Capitulum mitella* appears to have different life history patterns across the wide temperature gradient through physiological and developmental plasticity. For example, in Hong Kong (∼22°N), individuals reach sexual maturity within 9–12 months after settlement at a rostral-carinal length of approximately 14 mm, whereas in Fuzhou (∼25°N), maturity is reached after two years (Lin 1993; Leung 2003). Moreover, while only one annual settlement event is recorded in Fuzhou, multiple settlement events occur in Hong Kong (Lin 1993; Leung 2003). Given its broad latitudinal range, high thermal tolerance, and ecological adaptability, *C. mitella* represents an excellent model species for investigating responses to climate change at both genomic and transcriptomic levels (Yuan et al. 2024).

Mantel tests for the NW Pacific and SE Asian clade of *C. mitella* revealed that the genetic distance is not correlated with geographic distance. This is likely because larval dispersal in coastal barnacles is mediated by oceanic current patterns (Shahdadi et al. 2024). *Capitulum mitella* has a naupliar phase lasting approximately 10 days, during which larvae can tolerate a wide range of temperatures and salinities (Rao and Lin 2014, 2020). We therefore infer that the broad geographic range of the NW Pacific and SE Asian clade and genetic connectivity among localities from south of Vietnam to north of Japan are shaped by oceanographic regimes, including local and regional currents. For example, in the northern region of the South China Sea, there are several local surface circulations in different directions (Xue et al. 2004; Guan and Fang 2006; He et al. 2013; Liu 2013) which facilitate larval dispersal and genetic connectivity between Vietnam, Hainan, Hong Kong, and Taiwan. Further northward connections are likely facilitated by other regional currents such as the Taiwan Warm Current, the Min-Zhe Coastal Current, as well as the Kuroshio Current (see Liu 2013). Leung (2003) revealed larval supply from multiple geographical origins for *C. mitella* in Hong Kong, which further confirms the complex genetic connectivity in this clade.

Despite being under a single monophyletic clade, phylogeographic analyses detected a shallow and recent genetic divergence in the NW Pacific and SE Asian clade of *C. mitella*, representing two geographical populations. Localities in Honshu and South Korea are genetically well-connected and identified as the Northeastern population. Other localities from Zhoushan (China), Okinawa and Taiwan to Vietnam are also genetically well-connected and identified as the Southwestern population. Similar northern and southern population separations were recorded in the barnacle *Hexechamaesipho pilsbryi* (Tsang et al. 2013) and the intertidal oyster *Crassostrea ariakensis* (Li et al. 2021). Temperature and salinity are believed to be selection factors to affect the northern and southern genotype distribution of intertidal species in the NW Pacific (Li et al. 2021). Notably, the northernmost locality within the Southwestern population—Zhoushan, China—is situated just south of the Yangtze River mouth. The freshwater outflow from this river is recognized as a significant salinity biogeographic barrier, impeding reciprocal larval exchange between the northern and southern coasts for many coastal invertebrates (Dong et al. 2012; Chang et al. 2017). Glacial refugia can also be a factor affecting the Northeastern and Southwestern population distribution of the NW Pacific and SE Asian clade. Based on the current distributions, it is plausible that the Northeastern population originated from a glacial refugium in the Sea of Japan during the LGM, while the Southwestern population derived from a refugial population in the South China Sea. Following the LGM, both refugial populations underwent demographic and spatial expansion, giving rise to the present-day genetic and geographic patterns. Demographic analyses support this scenario, indicating expansions related to the LGM in both populations. BSP analyses further revealed that the Northeastern population, which inhabits more temperate and colder environments, expanded earlier than the Southwestern population. This earlier expansion is likely linked to the Northeastern population’s greater adaptability to cold climates (see above), enabling a faster recovery. Such recent demographic expansion is a widespread pattern in marine taxa and was also detected for all clades and populations of *C. mitella*, consistent with species’ responses to Pleistocene glaciation events (Tsang et al. 2012b; Ludt and Rocha 2014; Wu et al. 2015; present study).

Within the South clade, a genetic gap divides the clade into two groups: a Western group, broadly distributed from Bicol (southeastern Luzon) to Sabah (eastern Malaysia), and an Eastern group, primarily confined to Bicol. This shallow genetic divergence suggests that this split likely occurred during one of the Late Pleistocene glacial periods, when sea-level changes may have isolated the eastern coast of the Philippines from the western side (see Fig. 1D in Sholihah et al. 2021).

## Supplementary Material

obaf042_Supplemental_Files

## Data Availability

The specimens used in this study are deposited at biodiversity Research Center, Academia Sinica, Taiwan and are available with the codes given in the Table S1. The sequences are submitted to the GenBank, and are available under the accession numbers given in the Table S1.
