# Supplementary figures and images for: Genetic Structure of the Rocky Intertidal Stalked Barnacle Capitulum mitella Across the Northwest Pacific and Southeast Asia: Influences of Pleistocene Climate Changes and Contemporary Oceanographic Regimes
Source: Integr Org Biol. 2025 Nov 11;7(1):obaf042. doi: 10.1093/iob/obaf042 (PMC12699151; doi:10.1093/iob/obaf042)

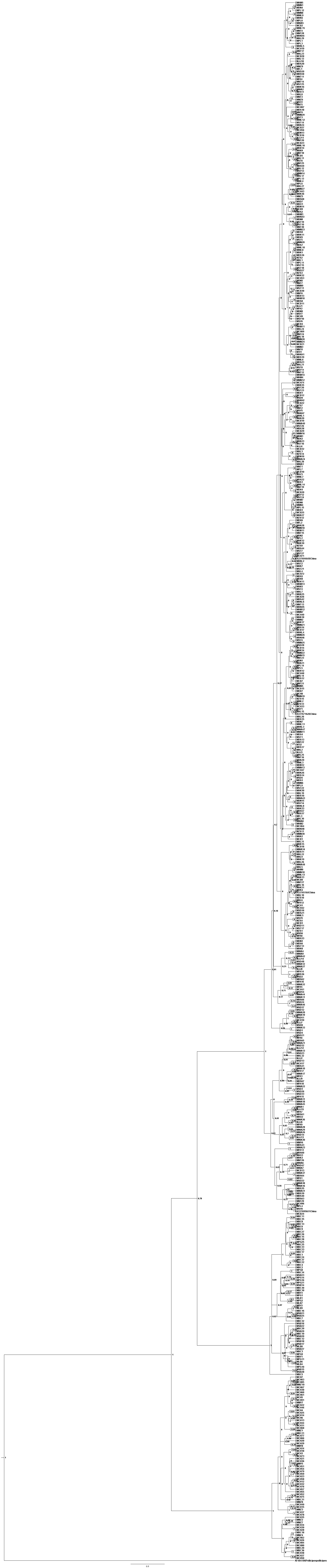

Supplement: obaf042_Supplemental_Files [file obaf042_supplemental_files.zip › Figure S1 BI tree full labels and supports.png]
